# It is not real until it feels real: Testing a new method for simulation of eyewitness experience with virtual reality technology and equipment

**DOI:** 10.3758/s13428-023-02186-2

**Published:** 2023-07-28

**Authors:** Kaja Glomb, Przemysław Piotrowski, Izabela Anna Romanowska

**Affiliations:** 1 Faculty of Management and Social Communication, Jagiellonian University in Krakow, Kraków, Poland; 2 Aarhus Institute of Advanced Studies, Aarhus University, Aarhus C, Denmark

**Keywords:** Eyewitness testimony, Methodology, Ecological validity, Immersion, VR

## Abstract

Laboratory research in the psychology of witness testimony is often criticized for its lack of ecological validity, including the use of unrealistic artificial stimuli to test memory performance. The purpose of our study is to present a method that can provide an intermediary between laboratory research and field studies or naturalistic experiments that are difficult to control and administer. It uses Video-360° technology and virtual reality (VR) equipment, which cuts subjects off from external stimuli and gives them control over the visual field. This can potentially increase the realism of the eyewitness's experience. To test the method, we conducted an experiment comparing the immersion effect, emotional response, and memory performance between subjects who watched a video presenting a mock crime on a head-mounted display (VR goggles; *n* = 57) and a screen (*n* = 50). The results suggest that, compared to those who watched the video on a screen, the VR group had a deeper sense of immersion, that is, of being part of the scene presented. At the same time, they were not distracted or cognitively overloaded by the more complex virtual environment, and remembered just as much detail about the crime as those viewing it on the screen. Additionally, we noted significant differences between subjects in ratings of emotions felt during the video. This may suggest that the two formats evoke different types of discrete emotions. Overall, the results confirm the usefulness of the proposed method in witness research.

**Supplementary information:**

The online version contains supplementary material available at 10.3758/s13428-023-02186-2.

## Introduction

For many decades, forensic experts have drawn attention to the limited possibility of reaching inferences about the real experiences of eyewitnesses based on the results obtained in laboratory studies. One of the most fundamental issues is the lack of ecological validity of such experiments (Chae, 2010; McKenna et al., 1992; Wagstaff et al., 2003; Yuille & Wells, 1991). During laboratory experiments, stimulus manipulation does not evoke states that mimic the experiences of real eyewitnesses. Participants who stay in a safe space are rarely surprised by stimuli, and do not confront unexpected events. Therefore, it is possible that their reactions to short films, slides, narratives, or recordings presenting a crime are the product of rational thought rather than instinctive responses. Thus, there is no shortage of voices in the literature encouraging more field research and analysis based on real crime cases (e.g., Yuille, 2013). This type of study, however, has its own challenges related to the limited ability to control for confounding variables and the need to rigorously repeat the procedure in situ, which is more complex and unpredictable (Grzyb & Doliński, 2021). Moreover, this type of research is demanding to organize and administer, which, with the heavy emphasis on increasing the sample size in psychological studies, can make it time-consuming and cumbersome (Doliński, 2018). As a result, the contribution of field or naturalistic experiments is very limited. In preparing this paper, we analyzed 1400 publications indexed in Google Scholar (search term: eyewitness testimony 'field study'), examining the abstracts of empirical articles and the method sections. We found that the vast majority of them are in one area of interest: the effects of alcohol and other psychoactive substances on witness memory. This may suggest that, for most psychologists, field experiments are a last resort, used essentially only when a safer, better-controlled laboratory alternative is not available.

With this in mind, the purpose of this study is to test a method that employs elements of virtual reality (VR) and its equipment for experimental manipulation. We believe that this procedure could provide an intermediate point between laboratory research and naturalistic or field experiments, as it allows exposure to more realistic stimuli. Empirical research in eyewitness testimony research already makes use of VR and Video-360° display equipment—for example, Kloft et al. (2020) in their study on false memory used virtual reality equipment and digital imagery. They simulated two criminal events in which the subjects played the role of an uninvolved witness of a physical attack on a policeman, or a perpetrator of theft in a bar. The scene was created with digitally generated graphics; thus the perpetrator, victims, and bystanders resembled game avatars. As we are not aware whether fully digital characters have the capacity to imitate humans in a way to produce effects similar to the experience of watching a real person being harmed, this type of manipulation will not necessarily be adequate for the study of emotions and phenomena typical of social situations. After all, as we know from the game research, one of the leading factors in determining how believable a so-called NPC (non-playable character) is depends on perceptual cues (e.g., Warpefelt, 2016)—thus, characters that look, move, express emotions, and behave unnaturally may not evoke similar psychological reactions as humans.

At this stage of the use of VR in eyewitness testimony research, however, the main obstacle is not so much the potential inadequacy of the stimuli, but rather a lack of methodological analysis of its effectiveness in inducing desired psychological states. Controlling for realism with few questions about the "realness" of the environment (e.g., Romeo et al., 2019), while important, does not allow us to fully determine the extent of immersion in the stimulus, and therefore subjects' engagement with the virtual world. Nor does it provide a way to identify these aspects of the method that can compete with more traditional research methods used in the psychology of witness testimony. We, therefore, decided to conduct a systematic study focused on VR, which appears to be essential to understanding the psychological states evoked by this medium. Our aim was to investigate the capability offered by virtual reality technology with respect not only to the realism of the experience but also to its potential consequences in terms of emotions and cognition.

To the best of our knowledge, this paper presents the results of the first methodological analysis of the potential of VR in eyewitness testimony research. Our study is set firmly in this field. While we do not ignore the body of work which demonstrates the capability of virtual reality to evoke emotions and arousal (e.g., Hofmann et al., 2021, who studied the subjective emotions and cortical α activity evoked by riding a rollercoaster in VR) or a sense of presence (e.g., Barreda-Ángeles et al., 2021, who used a design similar to ours while investigating journalistic pieces in terms of immersion and cognitive processing ), we believe that, with such a specific medium and research subject, it is essential to ensure that the particular context is addressed. For, as Yuille and Wells (1991) argue, in order for psychological lab research to be generalizable to real-life situations and to serve, for example, expert witnesses, it is essential to consider the contextual equivalence of the real eyewitness experience and the study. In our view, press materials and rollercoaster rides do not reflect this context; thus, our ability to infer the utility of VR in the paradigm of witness testimony is limited.

## Crucial limitations of laboratory experiments in eyewitness testimony

The discussion regarding the generalizability of laboratory research on memory has been ongoing for many decades, and any attempt to summarize it deserves a separate article. No less intense is the debate over the validity of laboratory research on eyewitness testimony—for some experts the overreliance primarily on laboratory studies is the reason for the deficient recognition of many psychological phenomena in the forensic field. An extreme position has been presented by Yuille (2013), who argues that *the context of the laboratory is so different from the context of many crimes, particularly violent crimes, that using the lab to study memory in the forensic context is pointless* (p. 9).

One key criticism of laboratory research on eyewitness testimony is that it often uses highly controlled and artificial stimuli, such as photographs or videos of staged events, rather than live events. These stimuli hardly apply to real situations, where witnesses often encounter more complex and dynamic stimuli for which they are not prepared. Processing of stimuli that are simplified or highly focused on a specific aspect of reality appears to be less prone to the distortions present with the high demands that crime observation places on the witness's real-life experience, even when their level of involvement is minimal. As a result, one can expect findings from lab-based research that suggest better witness memory performance than may be the case with higher distraction (Lane, 2006).

Other important aspects relate to the inability to simulate a sense of threat and fear in the lab, and the consequences (or lack thereof) that lab eyewitnesses suffer for making mistakes. However, from the point of view of this paper, the critique concerning the conditions for processing and encoding information is crucial—it is this lack of the naturalness of the stimulus that we are addressing with this research. With this in mind, the goal of our research was to verify an experimental method using VR elements to simulate the experience of an eyewitness. We believe that this method may overcome the limitations of typical witness testimony research, and has the potential to create a stimulus-rich, close-to-real experience, while maintaining high control and replicability of the procedure.

## How can virtual reality help experimental psychologists?

The definition of virtual reality is a subject of debate among experts, who do not always agree on the criteria that constitute VR. Since covering the discussion of this topic is beyond the scope of this paper, we focus solely on the criteria that justify the choice of this medium for psychological research. They relate primarily to the capacity users have in this environment and the degree of influence they have on it. Many experts would agree to use the term virtual reality only if the user has the ability to move, interfere, and change certain elements of that environment (Kardong-Edgren et al., 2019). For the purposes of this study, however, we adopted a less rigorous criterion: virtual reality is a digital space in which the user's movements are tracked, and their environment is continuously rendered and displayed according to those movements. Its purpose is to replace the signals coming from the real environment with digital ones (Fox et al., 2009). Therefore, a medium that adapts to the user's point of view and cuts off their access to real existing stimuli can be considered to be virtual reality.

These criteria are met by Video-360° (also called spherical video). Although the ability to influence the environment is limited to changing the field of view, the realism of this medium gives it an undeniable advantage over the strictly digital environment for psychological research. Video-360° uses recordings of real people in a real space. Therefore, researchers need not fear the effects that are present when it comes to realistic computer-generated characters (e.g., uncanny valley; Tinwell et al., 2011).

Some definitions of VR also focus not so much on the technology itself but place the user and their experience at the center. For example, as highlighted by Jerald (2015, p. 45): *VR is about psychologically being in a place different from where one is physically located, where that place may be a replica of the real world or may be an imaginary world that does not exist and could never exist*. This definition refers, not explicitly, to psychological phenomena reflecting a sense of presence, transportation, or immersion in a particular medium. They define the state of being absorbed by the environment, a sense of being part of it, and experiencing it (Rigby et al., 2019). These are related terms, but their meanings vary among those in the broad field of human-computer or human-media interactions. In this paper, we have chosen to use the term *immersion* primarily to ensure consistency between theoretical terms and research methods. It is crucial to underline that the immersion effect is, in our view, an index of the simulation's realism, and this is the focal point of this study, as by realism we consider not so much the accuracy of the reflection of some fragment of reality, but realism of the user's subjective experience. Similarly to Steuer et al. (1995), we believe that a sense of immersion can enhance the overall viewing experience, making it feel more real and lifelike. As a result, we can expect psychological states and behaviors similar to real life, as the medium is capable of invoking the illusions of place (a sensation of being in a real place) and plausibility (the illusion that the scenario being depicted is actually occurring) (Slater, 2009).

These main criteria—the ability for the users to change their point of view, isolation from external stimuli, and the capacity for immersion effect—are components of the simulation which better imitate the real-life experience of an eyewitness. However, these are not the only benefits of using VR in experimental procedures. It also automates the procedure so that it is consistent and not affected by external unexpected events (compared to staged crime). More complex systems also offer performance recording, which provides insight into what the subject is doing in this environment, e.g., via eye tracking. Thus, increased realism does not come at the expense of the rigor of the procedure or control of the experiment.

## Current study: Variables and hypotheses

Taking into account the nature of witness testimony research and, above all, the need to increase the ecological validity of the research while maintaining the rigor of the experimental procedure, we formulated the following hypotheses.

Our main dependent variable is immersion—an effect that can be described as being absorbed by a given medium (a game, a movie, or even a book). Thus, in this research, immersion is considered an operationalized realism of the experience. We expect that [H1] video watched on head-mounted displays (HMDs) creates a stronger immersion effect into the scene than a video watched on screen. The verification of this hypothesis is crucial for this study. If the participants have a higher sense of being present in the created scene and have the impression that they are in the space in which a crime is taking place, we would consider that the simulation has fulfilled its primary role, which is to increase the realism of simulation of the experience typical of an eyewitness. In addition to the main effect, we also expect differences in one of the subscale—Transportation. This subscale reflects a psychological state in which the distance between the observer and the scene is shortened, resulting in an observer feeling as if they are part of the events being presented. Achieving such a state seems to fulfill the previously mentioned definition of VR proposed by Jerald (2015), outlining a psychological "transfer" to a created reality.

A secondary issue with increased immersion relates to the consequences of this effect. As our objective was to develop a stimulus manipulation suitable for eyewitness testimony research, we assumed [H2] that subjects who watched the scene on HMD would feel stronger emotions than those who watched the same video on a screen. In particular, we expected higher negative emotions ratings accompanied by higher arousal. Therefore, we expected that our experiment would be in line with other studies suggesting an increased emotional response and arousal in VR (see, e.g., Estupiñán et al., 2013; Tian et al., 2021).

Due to the stimulus-rich environment, playing videos on head-mounted displays can also have negative consequences in terms of distraction and difficulty focusing on the scene presented. One of the challenges of creating any narrative in Video-360° format is to attract and direct attention to the focal actions, as the VR viewer has a much larger field of vision to explore (Dooley, 2017). As a result, participants in the experiment may ignore the events that are presented and focus on something completely different. Another problem related to immersive media such as VR is visual fatigue and cognitive overload, which can lead to impairment of certain cognitive functions (Frederiksen et al., 2020; Souchet et al., 2022a; for a review, see: Souchet et al., 2022b). In fact, there are some empirical studies suggesting the existence of this effect, although the material presented was much different from the one we prepared for this research (Barreda-Ángeles et al., 2021).

It is therefore necessary to examine whether attention processes—and the resulting memory processes—are in any way impaired in this stimuli-reach environment. As the purpose of the study was to test an experimental method suitable for research in eyewitness testimony, we chose long-term (episodic) memory as a measure of cognitive functioning. This type of memory is the main focus of research in this area. The theoretical and empirical rationale behind investigating the relationship between attentional processes and long-term memory is substantial. Prominent concepts in information processing recognize attention, working memory, and long-term memory as interconnected systems (for example, the embedded-process model proposed by Cowan, 1995,1999). Additionally, neuroscientific research provides evidence supporting the interaction between attention and long-term memory (for a review, see Chun & Turk-Browne, 2007). Hence, we set out to explore potential differences in event recollection. If the proposed simulation proved itself to be a valid research method, [H3] we would expect similar memory functioning in both groups.

## Materials and method

### Participants

A total of 115 subjects participated in the study (female = 76). Ultimately, due to incomplete questionnaires and device or recording malfunctions, 107 subjects (M_age_ = 22.18; SD_age_ = 2.74) were eligible for the final analysis. The experimental group (VR equipment) included 57 subjects (female = 38), while the control group (flat screen) included 50 subjects (female = 35). The groups did not differ in terms of age (*t*(104^1^) = .422; *p* = .674). As compensation for participation in the study, subjects were offered a 15-minute VR gaming session and an individual personality profile.

### Materials and apparatus

#### Experimental manipulation^2^

The video presenting a staged criminal incident prepared for the experiment was shot using Video-360° technology, which allows the full perceptual field to be observed. It lasts about three minutes and presents a scene in a pub with an outdoor garden. The criminal incident involves two perpetrators, male and female. They rob a girl who is sitting next to them. To carry out the theft, the male perpetrator turns to the victim and asks her for directions; at the same time, the female perpetrator approaches the table, takes a tablet and a wallet, and walks away from the scene. When the girl realizes that her belongings have been stolen and tries to run after the female perpetrator, the male stops her by pushing her onto a chair and knocking the rest of the items off the table.

#### Video display equipment

**Fig. 1:**
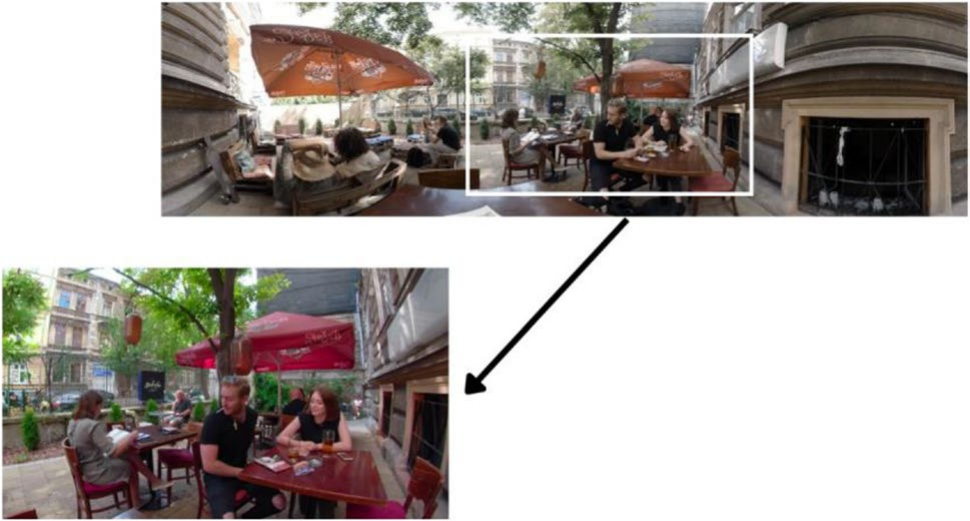
Comparison of perceptual fields accessible to subjects under two conditions. At the top is a 360-degree view as seen in HMD; the bottom screen shows the scene on a 2D screen.

We used an HP Omen laptop computer with a 15″ diagonal screen and HP Reverbs G1 goggles (head-mounted device). We used the HMD in the experimental conditions, and a computer screen in the control group. In the experimental condition, subjects were able to view the full perceptual field, covering 360 degrees, while the subjects watching the movie on the screen viewed a slice of that scene, covering the central visual field, which was adapted to the flat screen. A comparison of the image observed by subjects in both groups is presented in Fig. 1.

#### Post-event emotional ratings

The Geneva Emotion Wheel (GEW; Sacharin et al., 2012) was used to determine the valence and intensity of emotions experienced by participants while watching the film. This is a self-report measure consisting of discrete emotion labels corresponding to emotion domains that are arranged in a circle. The alignment of emotion terms is fundamental to the two-dimensional (2D) values (negative to positive) and control (low to high). The response options correspond to different levels of intensity for each emotion family, from low intensity (1) to high intensity (5). Subjects can also indicate that they did not feel a particular emotion (0), and they can independently label the name of the emotion they experienced.

#### Psychophysiological measurements

To assess arousal, we measured electrodermal activity (EDA). A wireless Shimmer3 GSR+ unit (worn as a wristband on the nondominant hand) and two EDA diodes were used. The unit was calibrated with a sampling rate frequency of 512 Hz. Subjects were asked to take a comfortable position, place their forearms on the desk, and attempt to minimize hand movement while watching the video. Preprocessing and further data analysis were performed in Python using a pyEDA (Hossein Aqajari et al., 2021).

#### Immersion assessment

To measure immersion in videos, we used the Immersive Experience Questionnaire for Film and TV (Film IEQ) developed by Rigby et al. (2019). The questionnaire was translated into Polish. It consists of 24 items and four factors: Captivation, real-world Dissociation, Comprehension, Transportation. The overall result of the questionnaire determines the strength of the immersion effect. Participants were asked to indicate on a seven-point scale how much they agreed with the statement.

#### Post-event memory performance

In this study, we analyzed episodic memory in delay condition and free recall procedure. For the purpose of the study, we created an index including the number of correctly remembered details about the event. The list of data that were considered includes information on the course of the event and the look and behavior of the perpetrators. It was developed by two competent judges who were unrelated to the project and not involved in the psychology of witness testimony. They were asked to watch a video (in the 2D variant) and then, immediately after watching it, to record all the information about the scene and the appearance and behavior of the people they watched. Based on the two lists we received, we created one covering all the noticeable details. We treated every detail as bits of information, which we then scored (if the information was given) in the subjects' responses. The maximum the subjects could report was 83 bits of information.

Due to differences between the videos in the size of the perceptual field, we only included information common to both conditions in the analysis.

### Procedure

The experiment was conducted in a between-subjects design. Subjects were randomly assigned to the experimental or control condition at the time of enrollment. The conditions differed in the type of equipment used to display the video. In the experimental conditions, subjects watched a 360-degree video played on head-mounted displays; in the control conditions, we used the traditional method of playing the video, i.e., on a flat screen.

Due to the health concerns of the subjects and the resulting sanitary rigor, the experiment was conducted in individual sessions. The whole procedure took about 1 h (+ c. 15–20 min for the game session offered as compensation). It included the following steps:

1. Preparation and baseline measurement (relaxing video) of electrodermal activity.
2. Exposure to stimulus (HMD versus flat screen) and electrodermal activity measurement.
3. Emotion self-report. Immediately after watching the video, subjects were asked to rate the intensity of emotions they felt while watching the video. We wanted to measure them soon after the film ended, so that the emotions would still be vivid and could be evaluated easily.
4. Immersion measurement (self-report).
5. Filler task designed to delay the memory testing, allowing us to study long-term memory rather than working memory. The participants completed questionnaires, the results of which will not be reported here.
6. Free recall memory task. Respondents were asked three questions: (1) *Tell all you remember about the scene in a pub that you just watched, both about how the scene unfolded and about the people who participated in it*. (2) *Do you remember anything about the appearance of the main characters*? (3) *Is that all you remember about the film? *The task format, i.e., including three questions, was developed after the pilot study which showed that subjects, when asked to describe "everything they remember," were limited to a very schematic and brief description of the events. As very short description do not allow for a reliable comparative analysis, we decided to expand the task and ask three questions. As our study is concerned with eyewitness testimony, the question about the perpetrators' look was crucial (this type of information is often collected by investigators to identify the perpetrators.). We also added a third question in case that a subject remembered something about the perpetrators' behavior after recalling their appearance. Subject responses were recorded using a voice recorder. The recordings were then transcribed and coded to be analyzed in terms of the amount of information provided. The time interval between the encoding memory and recollection was set at 25 min.

The procedure was positively reviewed and approved by the Research Ethics Committee at the Institute of Applied Psychology at the Jagiellonian University before its application (decision number 56/2019 dated 25 November 2019).

## Results

For statistical analysis, we used PS Imago (IBM SPSS Statistics 28), JASP 0.16.4.0, and Python 3.10. The default software was SPSS; thus, we only specify when the analyses were performed with different tools.

### Hypothesis 1. Videos watched on HMD are more immersive than those watched on screen

Our main objective was to verify the hypothesis of deeper immersion of a video viewed on HMD. To examine this, we used Film IEQ to measure the overall immersion effect and its components. We were most interested in the main effect, but we also expected to see a difference in terms of Transportation. The results of the subjects’ ratings and between-subjects comparison are presented in Table 1.

**Table 1:** Rates of immersion measured with Film IEQ with between-subjects comparison (N = 107)

	Condition	*M*	*SD*	Between-subjects *df =* 105
**Immersion** (main effect)	**VR**	**4.87**	**.592**	***t*** **= 2.756;** ***p*** **= .003;** ***d*** **= .534**
	Screen	4.56	.55	
**Captivation**	**VR**	**5.12**	**.799**	***t*** **= 2.963;** ***p*** **= .002;** ***d*** **= .574**
	Screen	4.71	.623	
Dissociation	VR	4.94	1.481	*t* = .132; *p* = .189
	Screen	4.71	1.148	
Comprehension	VR	4.67	.759	*t* = –.553; *p* = .291
	Screen	4.75	.797	
**Transportation**	**VR**	**4.36**	**1.044**	***t*** **= 1.963;** ***p*** **= .026;** ***d*** **= .380**
	Screen	3.96	1.087	

Due to directional hypotheses, assuming stronger immersion in the experimental condition, we report one-tailed significance.

Bold indicates statistically significant differences (one-tailed *p*) between conditions.

A comparison made using a one-tailed (given the directional hypothesis) *t*-test for independent samples showed that participants who watched the video on HMD rated immersion (*t*(105) = 2.756; *p* = .003; *d* = .534) and its two components, Captivation (*t*(105) = 2.963; *p* = .002; *d* = .574) and Transportation, higher (*t*(105) = 1.963; *p* = .026; *d* = .380). Ratings for the two other factors, namely Comprehension (*t*(105) = –.553; *p* = .291) and Dissociation (*t*(105) = .132; *p* = .189), did not differ between conditions. Given that we primarily expected a significant difference in the main effect, we consider Hypothesis 1 to be confirmed.

### Hypothesis 2. Video watched on HMD evokes stronger emotions and higher arousal

Our second hypothesis relates to the potential consequences of the immersion effect, i.e., stronger emotional responses. In this experiment, we examined subjects’ rates of emotions in terms of their intensity and valence, as well as psychophysiological arousal. The first two aspects were examined using self-reports (GEW), while arousal was operationalized as electrodermal activity (EDA).

#### Post-event emotional self-ratings

To answer the question of whether video played on HMD evokes stronger emotions than video played on a screen, we analyzed the answers that subjects gave in the GEW. We first analyzed all discrete emotion labels and compared them between conditions (Table 2). The analysis indicates that the only emotion that the subjects in the experimental group (VR) rated higher was guilt (*t*(79.53*) = 2.753; *p* = .004; *d* = .520; one-tailed significance). Moreover, contrary to our directional hypothesis, participants who watched the video on the screen rated hate (*t*(89.69*) = –2.368; *p* = .010; *d* = .455) and anger *t*(104.97*) = –2.928; *p* = .002; *d* = .562) higher. These emotions, rather than fear, are expected after watching a criminal incident (theft and assault), as the subjects were not at risk of any harm.

**Table 2:** The rates of emotion assessment (GEW) reported by subject and between subjects comparison (N = 107)

	Condition	*M*	*SD*	*SE*	Between-subjects
Sadness	VR	.84	1.251	.166	*t*(93.45*) = –1.365; *p* = .088/.169**
	Screen	1.22	1.569	.222	
**Guilt**	**VR**	**.77**	**1.268**	**.168**	*t*(79.53*) = 2.753; ***p =*** **.004/.007;** ***d =*** **.520**
	Screen	.26	.565	.080	
Regret	VR	1.61	1.623	.215	*t*(105) = –.346; *p =* .365/.730
	Screen	1.72	1.526	.216	
Shame	VR	.91	1.550	.205	*t*(105) = –.635; *p* = .264/.527
	Screen	.74	1.209	.171	
Disappointment	VR	1.42	1.742	.231	*t*(105) = –1.428; *p =* .078/.156
	Screen	1.90	1.717	.243	
Fear	VR	1.68	1.502	.199	*t*(105) = 1.278; *p* = .102/.204
	Screen	1.32	1.435	.203	
Disgust	VR	1.46	1.794	.238	*t*(105) = –285; *p =* .280/.560
	Screen	1.66	1.803	.255	
Contempt	VR	2.11	1.790	.237	*t*(105) = –1.400; *p* = .082/.164
	Screen	2.58	1.703	.241	
**Hate**	**VR**	**.491**	**1.020**	**.135**	*t*(89.69*) = –2.368; ***p =*** **.010/.020;** ***d*** **= .455**
	**Screen**	**1.04**	**1.370**	**.194**	
**Anger**	**VR**	**2.04**	**1.792**	**.237**	*t*(104.97*) = –2.928; ***p =*** **.002/.004;** ***d*** **= .562**
	**Screen**	**2.98**	**1.545**	**.219**	
Interest	VR	3.05	1.747	.231	*t*(105) = .593; *p =* .277/.554
	Screen	2.86	1.591	.225	
Amusement	VR	.46	.946	.125	*t*(105) = .425; *p =* .336/.672
	Screen	.38	.901	.127	
Pride	VR	.02	.132	.018	*t*(105) = .935; *p =* .176/.351
	Screen	.00	.000	.000	
Joy	VR	.21	.674	.089	*t*(105) = –.583; *p =* .281/.569
	Screen	.30	.909	.129	
Pleasure	VR	.91	1.573	.208	*t*(104.45*) = 1.348; *p =* .090/.180
	Screen	.54	1.281	.181	
Contentment	VR	.40	1.033	.137	*t*(104.30*) = 1.127; *p =* .134/.269
	Screen	.20	.833	.118	
Love	VR	.02	.132	.018	*t*(105) = –.536; *p =* .296/.593
	Screen	.04	.283	.040	
Admiration	VR	.12	.600	.079	*t*(93.11*) = –1.197; *p =* .121/.241
	Screen	.28	.757	.107	
Relief	VR	.16	.560	.074	*t*(105) = –.187; *p =* .426/.852
	Screen	.18	.661	.093	
Compassion	VR	2.67	1.806	.239	*t*(105) = –.536; *p =* .113/.226
	Screen	3.08	1.688	.239	

* Equal variances not assumed.

** As some of the results turned out to be significant but contrary to our hypothesis, we report the significance of both one-tailed and two-tailed tests. Bold indicates statistically significant differences between conditions at least in a one-sided test.

In the second step, we created general indices of domains of emotions, in line with the theoretical background of the method (Scherer, 2005). Each indicator is an averaged rating of an emotion belonging to one of the quarters of the GEW (negative valence, low control; negative valence, high control; positive valence, low control; positive valence, high control). As can be seen in Table 3, there is a significant (one-tailed) difference (*t*(105) *=* –1.762; *p* = .040; *d* = .349) between conditions with respect to the ratings of emotions with negative valence and high control. This is a consequence of higher ratings for anger and hate that comprise this domain. However, significantly, the result is opposite to the one we assumed.

**Table 3:** The average results of emotions indices in each study condition and the between-subjects comparison (N = 107)

Emotions domains	VR *M* (*SD*)	Screen *M* (*SD*)	Between subjects comparisons *df* = 105
NE	1.33 (.95)	1.54 (.81)	*t =* –1.218; *p* = .113/.226*
**NE high**	**1.55 (1.08)**	**1.92 (1.04)**	***t =*** **–1.762;** ***p*** **= .040/.081;** ***d*** **= .349**
NE low	1.11 (1.06)	1.17 (.93)	*t =* –.288; *p* = .387/.774
PE	.80 (.46)	.78 (.43)	*t =* .181; *p* = .428/.857
PE high	.93 (.67)	.82 (.63)	*t =* .905; *p* = .184/.368
PE low	.67 (.50)	.76 (.47)	*t =* –.874; *p* = .192/.384

*As some of the results turned out to significant. However, contrary to our hypothesis. we report the significance of both one-tailed and two-tailed tests.

NE: Negative Emotion index; NE high: negative valence–high control index; NE low: negative valence–low control. PE: positive Emotion index. PE high: positive valence–high control; PE low: positive valence–low control.

Bold indicates statistically significant differences between conditions at least in a one-sided test.

#### Psychophysiological measurements

**Fig. 2:**
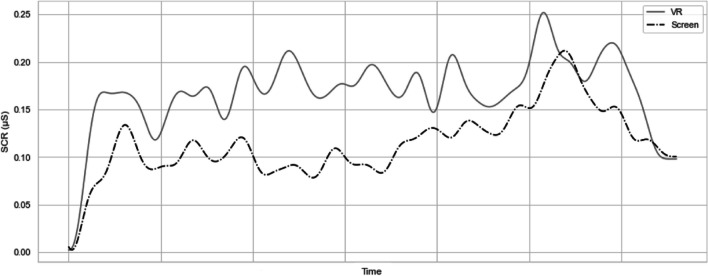
Filtered electrodermal response recorded while watching the video. Between-subjects comparison

We began the EDA analysis by checking the data for any recording errors or artifacts that might strongly distort the measurement. As we did not identify such records, we performed the analysis using the calculation method proposed by Hossein Aqajari et al. (2021). First, we examined the mean level of electrodermal activity recorded when subjects watched the video. This allowed us to determine the overall arousal induced by the medium. Figure 2 presents the filtered electrodermal activity. To eliminate individual differences in perspiration, we compared the measurements recorded during the crime video with baseline measurements recorded during the preparation for the experiment. We compared two segments lasting 165 seconds, omitting the first seconds of the video because of the potential novelty effect that may cause arousal.

Table 4 presents the results of our analysis (“mean activity”). Although we were unable to obtain a significant difference (*t*(105) = 1.553; *p* = .062) between the conditions in the one-tailed test (owing to the directional hypothesis assuming higher arousal in the experimental condition), we can describe these results as on the verge of significance.

**Table 4:** Summary of electrodermal activity analysis (N = 107)

	Condition	Mean activity (165 s.)	Max. peak (18 s.)	*M*	*SD*
		*M*	*SD*		
Baseline	VR	1.86	1.72	-	-
	Screen	1.50	.92	-	-
Film	VR	2.78	2.74	3.06	3.01
	Screen	2.13	1.48	2.34	1.76
Difference (film – baseline)	VR	.93	1.14	-	-
	Screen	.63	.74	-	-

*Note*: Values are provided in microSiemens (μS).

The second step of our analysis was to compare only the end of the video—that is, the several seconds (18 s) during which the crime occurred. This is because we wanted to isolate the arousal caused by the crime stimulus itself, not the entire video. The results are presented in Table 4 (“max. peak”). To verify the hypothesis of stronger arousal experienced when the crime itself was observed on the HMD rather than on the screen, we compared the maximum amplitude peak between conditions. Once again, we observed close to significance in a one-tailed *t*-test (*t*(91.98) = 1.529; *p* = .065). To summarize the analyses performed to verify Hypothesis 2, we can cautiously conclude that subjects under the experimental conditions were more aroused than those in the control group. At the same time, they generally rated negative emotions with high control lower than those who watched the film on the screen. However, they felt stronger guilt than the subjects in the control condition. Thus, we consider these results to be inconclusive.

### Hypothesis 3. Video displayed in VR HMD is not more distracting than video played on screen

We considered post-event memory performance as a measure of distraction. We assumed that distraction would be indicated by a lower number of correctly reported pieces of information about the crime scene. Thus, to compare recollection between conditions, we used an index covering the number of details accurately remembered by the subjects. Table 5 presents the results. The *t*-test revealed that the conditions do not differ in the number of correctly remembered details (*t*(105) = .073; *p* = .942). However, as our hypothesis stated that there are no differences in recollection, we also decided to use Bayesian statistics and to apply the Bayes factor (BF) in the interpretation. BF is interpreted as the ratio of the probability of obtaining given observations in two comparable models (null hypothesis and alternative hypothesis; Masson, 2011). We performed the analysis in JASP and adopted the interpretation of the factor according to Andraszewicz et al. (2015): BF1–3 = anecdotal evidence for the null hypothesis; BF3–10 = moderate evidence for the null hypothesis; BF10–30 = strong evidence for the null hypothesis. The Bayesian independent *t*-test shows that there is moderate evidence for H0 (that is, there is no difference in terms of the number of correctly recalled bits of information about the event, BF_01_ = 4.866).

**Table 5:** Number of pieces of information about the crime event correctly recalled (N = 107)

							95% CI	
	Condition	N	*M*	*SD*	*SE*	*CV*	Lower	Upper
Recollection	VR	57	20.60	7.01	0.93	*0.340*	18.74	22.46
	Screen	50	20.50	6.59	0.93	*0.322*	18.63	22.37

In addition, to investigate more subtle aspects of recollection, we also analyzed misreports. We took into account both types of errors: (1) *distortions*, which are all bits of information that involve details that were present in the video but were incorrectly reported (e.g., incorrect color of pants, misremembered behavior), and (2) *additions*, which are all the bits of information that were absent in the video but were reported by subjects. As can be seen in Table 6, the mean number of both types of errors, but also their overall value (Σ distortions + additions), is similar in VR and Screen conditions. Between-subjects comparison also showed no statistical difference in the number of errors; however, in the case of distortions there is only anecdotal evidence for the null hypothesis (BF_01_ = 2.46). Finally, we decided to investigate the overall accuracy of recollection and compare the rates between conditions. We define recollection rate after Evans and Fisher (2011) as the number of accurately provided details (see Table 5) of the event / Σ accurate + errors (see Table 6). The rates, as shown in Table 7, are almost identical for both conditions, and between-subject comparison indicates that there are no differences in terms of the accuracy of the recall (*t*(105) = .127; *p* = .899). The Bayesian *t*-test provides moderate evidence for the null hypothesis (BF = 4.84). Considering all the above analyses performed for Hypothesis 3, we conclude that it has been confirmed.

**Table 6:** Number of errors in recollection (N = 107)

						95% CI		Between-subject comparison
	Condition	*M*	*SD*	*SE*	*CV*	Lower	Upper	
Distortions	VR	.39	.77	.102	2.00	.19	.59	*t*(105) = 1.240 ; *p* = .280 BF_01_ = 2.46
	Screen	.22	.58	.082	2.64	.06	.38	
Additions	VR	2.47	1.42	.187	.57	2.10	2.84	*t*(105) = –.272 ; *p* = .786 BF_01_ = 4.72
	Screen	2.54	1.05	.149	.415	2.25	2.83	
Total errors	VR	2.86	1.62	.215	.566	2.44	3.28	*t*(105) = .352 ; *p* = .725 BF_01_ = 4.61
	Screen	2.76	1.26	.177	.455	2.41	3.11	

*Note*: Distortions are all bits of information that involve details that were present in the video, but incorrectly reported (e.g., incorrect color of pants, misremembered behavior).

Additions are all the bits of information that were absent in the video but reported by subjects.

* Due to the violation of equal variation assumption, a Welsh *t*-test with Satterthwaite approximation for the degrees of freedom was used.

**Table 7:** Accuracy rates of recollection (N = 107)

				Between-subjects comparison
	Condition	*M*	*SD*	
Accuracy Rates	VR	.877	.063	*t*(105) = .127; *p* = .899 BF_01_ = 4.84
	Screen	.875	.069	

*Note*: The accuracy rate is defined as the number of accurately provided details (see Table 5) of the event / Σ accurate + errors (see Table 6).

## Discussion

In the experiment (*N* = 107) in which we compared two types of video display devices (head-mounted device and flat screen), and thus two formats of video recording (Video-360° and 2D video), we obtained results suggesting that our proposed method may be a more realistic alternative to traditional stimulus manipulations using videos. We infer the higher realism of the subjects' experiences primarily based on the difference in terms of immersion effect evoked during stimuli manipulation. We observed higher rates of immersion and its two factors (Captivation and Transportation) among people who watched the video on HMD; thus, we believe that this medium offers researchers the potential to elicit in subjects a sense of being highly engrossed in a mediated experience. Our results suggest that the VR group felt more involved in the video and were more motivated to watch it (Captivation). Furthermore, there are some arguments in favor of the notion that, while watching a criminal incident on HMD, subjects felt like they were experiencing events for themselves and were located in the world portrayed in the video (Transportation). These differences between conditions indicate that the proposed method increases the realism of the experience and shortens the distance between the observer and the scene.

The results of our study can be related to the concept of two different types of realism in laboratory research introduced by Aronson and Carlsmith (1968) and developed by Wilson et al. (2010). The researchers proposed to assess lab research in terms of experimental and mundane realism. The first one implies subjects’ involvement in the situation created in the laboratory and the authentic experiences evoked during the task, while the latter is defined as the similarity of the experimental situation to events that might happen in real life. The results of our study support the argument that VR may enhance both types of realism. On the one hand, subjects in the VR group were more engaged in the experiment, as evidenced by higher scores in Captivation; on the other hand, they felt as if they were part of the crime event (Transportation), which appears to satisfy the definition of mundane realism. Therefore, we believe that studies that use VR for stimulus presentation seem to be less burdened by the accusation that is made against traditional laboratory research in eyewitness testimony, which points to the “artificiality” of experimental manipulation.

In contrast to immersion, we obtained inconclusive results when comparing subjects' emotional responses between conditions. On the one hand, we can argue (with some caution) that subjects in the experimental conditions were slightly more aroused than those in the control conditions, although the results are only on the cusp of significance in one-sided tests. To evaluate the level of uncertainty associated with the results, we conducted additional analyses in which we used bootstrapping simulation. Their results (see Appendix—Supplementary Analysis B) provide additional support for the notion that the subjects' arousal was higher while watching the crime scene in VR than on the screen. First, parametric bootstrapping (resampling 10,000 times) demonstrated a significant difference between the conditions in terms of the change in arousal between the baseline measurement and the arousal experienced during the viewing offense. Secondly, the permutation test showed that although the maximum arousal registered during the last scene (the actual crime) was comparable, this finding is only true for low and medium amplitudes. For the most responsive subjects, the crime scene viewed in VR was significantly more arousing than the scene presented on the screen. These results suggest that experimental manipulation in VR may be recommended in particular for a strong emotional stimulus and/or a population with a low arousal threshold. Our study thus indirectly supports the finding of Slater et al. (2006), who showed a significant increase in arousal in an anxiety situation experienced in VR in phobic-sensitive subjects.

On the other hand, we obtained rather surprising results in ratings of the intensity of discrete emotions. They indicate stronger anger and hate felt by the subjects in the control conditions and more intense guilt felt by those in the experimental group.

First, it is necessary to address the discrepancy between the two measurements of the components of emotion (subjective feeling and psychophysiological measurement). This inconsistency is explainable theoretically, and parallels in other empirical studies can be pointed out (e.g., Mauss et al., 2004; Chivers et al., 2010; Ciuk et al., 2015). People struggle to identify and evaluate the intensity of emotions for various reasons. Labeling a specific emotion may be difficult, as during the emotional process they may quickly change (Scherer, 1987). Moreover, some stimuli may elicit emotions that are more complex and multifaceted than those captured by simple measurement methods associated with discrete emotions.

However, this discrepancy does not explain why subjects watching the crime in VR felt stronger guilt, while those watching the video on screen rated anger and hate higher. As our research is the first attempt at methodological analysis of stimulus manipulation using Video-360° and VR equipment to compare discrete emotions, in discussing the results, we decided to include two alternative explanations—we consider them as a starting point for future research on the proposed technique in witness testimony studies.

First of all, it should be considered that such emotion ratings adequately represent the subject's emotional experience, and therefore, in fact, these two media elicit different kinds of emotion. Based on the theory of emotions, it is possible to formulate possible explanations of which aspects of the experimental manipulation may be considered as their antecedents.

### Guilt

Although we commonly think of shame and guilt as feelings we experience as a result of our own actions, feelings of self-condemnation can sometimes result from acts committed by others. In such a situation, we can refer to so-called *vicarious guilt*, as Lickel et al. (2005) defined it. It assumes that personal causality is not always a prerequisite for the experience of guilt, but that there are certain conditions that may induce it. Thus, referring to Lickel's research, it seems possible that subjects who experienced increased transportation to the crime event and immersion in the scene could have felt stronger vicarious guilt due to virtual reality-induced control of the situation. Perhaps they felt while watching the crime that they could have done something—helped the victim catch the perpetrators, or even stopped them before the crime occurred. Importantly, the intensity of this guilt is not high. This may be because the emotion was triggered by the behaviors and actions of someone else, not themselves. This explanation, however, needs further verification with methods capable of discriminating between different types of guilt.

### Anger and hate

These two emotions are substantively content related and are sometimes considered together (e.g., Bernier & Dozier, 2002; Frijda, 1986; Power & Dalgleish, 2015). Anger is often defined as a modal/basic emotion. By signaling significance at the individual–environment interface, it organizes a response to the stimulus, which often takes the form of aggression. However, anger is not necessarily a response to a stimulus directly related to the individual's self, but can also be triggered by aversive environmental stimuli, such as unpleasant sights, smells, and extreme thermal sensations (Berkowitz, 1990). In this sense, then, it appears more similar to hate than to a modal emotion that prepares for a fight. After all, one way to understand hate—on an individual, not a group level—is to define it as a strong feeling of intense or passionate dislike for someone or something. When considering hate, we most commonly refer to an emotion aroused by frustration of needs or an unpleasant sensory experience (Brogaard, 2020), but this emotion also has links to the moral evaluation of certain behaviors (Pretus Gomez et al., 2022). In this sense, hate and anger are emotions that could be evoked by the video that presents two individuals committing a crime and behaving in an irritating manner. Juxtaposing self-report rates with psychophysiological measurements (lower arousal in control condition), we can conclude that the video probably did not evoke violent, highly arousing emotions or trigger a fight/flight response. It results rather in a moral emotion based on evaluation of the culprits’ behavior. This line of reasoning, however, requires additional research that would provide a more in-depth understanding of the subjects' emotional states. Methods based on a free response format (e.g., Geneva Affect Label Coder; K. R. Scherer, 2005) or focused interview may be most useful.

However, why these emotions were felt more intensively when the crime was seen on the screen is more challenging to explain. Perhaps this format allowed the subjects to focus more on the course of the events they were watching. They had no influence on the visual field, so they could only follow what the perpetrators did. As a result, their attitude, conversation, and actions may evoke stronger emotions. Such an explanation would be consistent with research results indicating that shifting attention from an emetogenic stimulus to its background significantly reduces emotional experience (e.g., Dolcos et al., 2020). Moreover, the ability to change attention is one of the theoretical factors mediating emotional experience: it is necessary for regulating emotions and, therefore, maintaining desirable emotional states (Wadlinger & Isaacowitz, 2011).

This format-driven focus exclusively on a key part of the scene, reducing the ecological validity of the "witnessing" experience, may have also made the scene less ambivalent and simpler to interpret. Meanwhile, the scene viewed on HMD gave the subjects some control over the experience—although they remained static (they couldn't change their seats), they could look away, and see how others were reacting. Perhaps, too, the incident was more surprising or startling, which was not covered by the self-report method we used. As a result of being present in the space with other eyewitnesses, the subjects' responses may have been influenced by how other people present in the pub behaved (the characters expressed surprise and incomprehension of what had happened—both with their reactions and verbally). After all, as Erber and Erber (2000) stated, people are often compelled to regulate affective states according to the demands of the situation, and social appropriateness (especially when interacting with strangers) is one of the most prominent motives for self-regulation. Thus, in the experimental conditions, perhaps emotions more appropriate to the situation were evoked, not so much anger and hatred toward the perpetrators, but surprise that the robbery happened at all. However, this interpretation requires verification to determine the intensity of the surprise felt in a VR environment.

An alternative explanation for the results obtained in the study can be offered. It refers not so much to the results of the control group, as to the experimental one. Researchers investigating the immersion effect, in particular the presence in a virtual environment, draw attention to the essential hedonic nature of this experience. As Murray (2017, p. 98) states, “*The experience of being transported to an elaborately simulated place is pleasurable in itself, regardless of the fantasy content*.” Accepting this explanation, it can be argued that this pleasurable nature of being in a simulated space among other people on a warm, summer day may have resulted in the suppression of negative emotions in the experimental group. This explanation is all the more plausible when one considers that the study took place during a period of a sanitary regime (related to the COVID-19 pandemic), which limited opportunities for social participation^3^. On the other hand, the results of the comparison of conscious positive emotions do not differ between the groups. However, subjects who watched the video on HMD rated it slightly higher than those who watched it on a screen (M_VR_ = .91, SD = 1.57; M_screen_ = .54, SD =1.28; *t*(104.45) = 1.348; *p* = .090 (one-tailed).

Our study also demonstrated that the proposed simulation method does not affect memory processes. This indicates that a full Video-360° stimulus environment is not more distracting, nor does it lead to cognitive exhaustion. Thus, our research does not confirm the results obtained by Barreda-Ángeles et al., 2021, who observed that a virtual reality environment can harm focused attention, recognition, and cued recall of information. This discrepancy is likely due to a significant difference in the content presented. While our study tried to present a realistic crime scene, and therefore a video that can be used in the psychology of eyewitness testimony, the study by Barreda-Ángeles et al. used journalistic excerpts, with specific narration and editing. While virtual reality can cause cognitive fatigue in situations where the task is also performed in this environment, it is multimodal in nature, and the quality of the simulation causes negative phenomena such as simulator sickness or visual sense interference (Nash et al., 2000; Souchet, et al., 2022b), we believe that our Video-360° was easy to process. The scene presented in this research appears to be realistic, coherent, and thus processed fluently—it is not so much a content carrier, but more of a presence in the environment itself. However, the scene in VR directs attention and forces concentration on the elements chosen by the developer, so in this respect it is still a proxy of the witness experience, in which case greater memory disruption is expected (Ihlebæk et al., 2003).

### Limitations and future research

Although our study identified that the potential application of virtual reality in memory research is an important contribution to the research methodology in the psychology of eyewitness testimony, it is not free from limitations. As the study compared a video presented on-screen with one mediated by virtual reality equipment, the ability to infer the ecological validity of this method is still limited. For a method to be considered more ecologically accurate, a comparison with a natural experiment is necessary. Nevertheless, based on the results, we can infer a higher realism of the witnesses' experience—a deeper sense of being a real observer of the crime, rather than a viewer of a crime film.

Another limitation is the relatively modest sample size, which probably resulted in some of the analyses not yielding significant results and being only on the verge of significance. However, the research was conducted during a period of sanitary regime, which not only made it harder to access potential participants, but also slowed the research process. Research using virtual reality equipment required subjects to be present in the laboratory and could not be carried out over the Internet. Therefore, we decided to conduct the experiment within the scheduled project period, even at the cost of a smaller sample size.

We believe that the research should be repeated not only due to the small sample size but also to the surprising results of the emotional response analysis that are contrary to the hypotheses. Given that the tool we chose to measure the intensity of emotions did not allow us to capture surprise, we are not certain that the idea of the coherence between the reactions of the subjects and other “eyewitnesses” presented in the film is adequate. Future research should therefore compare the reaction of being startled. This would provide a stronger argument that the behavior and reactions elicited by the simulation using VR are realistic. Optimally, though, similar comparisons should be made between the VR experiment and the naturalistic one. Moreover, a more in-depth analysis of the subjects' emotional states and experiences of observing the crime is also necessary—ideally, one that allows the subjects to describe their states without the researcher's suggestion of how to label them. The account of more complex phenomenological experiences can potentially be compared to actual witnesses' emotional states, and this may provide a key argument for recognizing the proposed method as a valid simulation of witnesses' experiences.

Furthermore, the potentially pleasant nature of VR-mediated experiences should also be verified. As mentioned above, one possible explanation for the lower ratings of negative emotions in VR may be their suppression by the pleasurable nature of virtual reality. To determine whether an experimental manipulation mediated by VR in fact evokes different emotions than one performed using a traditional method, it is necessary to repeat the experiment during the period of ordinary access to social life. Another way to test it is to prepare a different stimulus that is not as easily associated with pleasure and leisure.

Given the above, however, we believe that our study represents an important step in the development of an ecologically valid experimental method. It can potentially change not only the psychology of witness testimony, but also more general studies of other mental function or behavior, so that they are set in a more realistic context without losing control of the procedure.

### Supplementary Information

ESM 1 (DOCX 1595 kb)

## Footnotes

1 One person in the experimental group did not report age.

2 The video has been deposited in a repository and is available for noncommercial use by researchers under a CC BY-NC-ND 4.0 license. DOI: 10.26106/r0av-bn42 https://ruj.uj.edu.pl/xmlui/handle/item/308227?locale-attribute=en

3 We consider this explanation plausible also in light of the qualitative assessment of the subjects' reports, which they spontaneously produced after watching the film. A portion of the participants, when asked to describe the scene, also highlighted their own emotions and described their experience. Some statements included accounts of the pleasure they felt during the simulation. Since description of emotional states in free response format was not part of the procedure, we did not systematically analyze them, but we consider this explanation plausible and in need of verification by more sensitive methods.
